# Latent tuberculosis infection among adults attending HIV services at an urban tertiary hospital in Malawi

**DOI:** 10.1097/QAD.0000000000003359

**Published:** 2022-11-23

**Authors:** Steven C. Mitini-Nkhoma, David T. Mzinza, Elizabeth T. Chimbayo, Aaron P. Chirambo, David V. Mhango, Cheusisime Kajanga, Christine Mandalasi, Dumizulu L. Tembo, Jane Mallewa, Leo Masamba, David G. Russell, Kondwani C. Jambo, S. Bertie Squire, Henry C. Mwandumba

**Affiliations:** a Malawi Liverpool Wellcome Clinical Research Programme, Kamuzu University of Health Sciences; b Department of Medicine, Kamuzu University of Health Sciences and Queen Elizabeth Central Hospital, Blantyre, Malawi; c Microbiology and Immunology, College of Veterinary Medicine, Cornell University, Ithaca, NY, USA; d Department of Clinical Sciences, Liverpool School of Tropical Medicine, Liverpool, UK.

Active tuberculosis (TB) can develop from newly acquired *Mycobacterium tuberculosis* infection or from reactivation of pre-existing latent TB infection (LTBI). Although individuals with LTBI do not have clinical TB symptoms, they are a reservoir for active TB cases and pose a significant challenge to the global effort to eliminate TB. Strategies to reduce the burden of TB vary among countries, and are, in part, determined by the local prevalence of LTBI and active TB [1]. Developing such strategies for sub-Saharan African (SSA) countries is challenging because of the paucity of data on the prevalence of LTBI in the region [2]. To address this knowledge gap, we conducted a cross-sectional study to determine the prevalence of, and factors associated with LTBI among asymptomatic people with HIV (PWH) and healthy, HIV-uninfected adults attending antiretroviral therapy (ART) and HIV voluntary counselling and testing clinics at Queen Elizabeth Central Hospital, a large urban tertiary hospital in Blantyre, Malawi.

All study participants were Malawian adults aged at least 18 years who had received Bacillus Calmette-Guérin vaccination during childhood but had no history of previous TB treatment or clinical and laboratory evidence of active TB. They were tested for LTBI using the QuantiFERON-TB Gold Plus (QFT-Plus) interferon gamma release assay (IGRA) (QIAGEN, Hilden, Germany) according to the manufacturer's instructions. Informed written consent was obtained from all participants and the study protocol was approved by the College of Medicine Research Ethics Committee in Malawi (protocol P.02/18/2356) and the Liverpool School of Tropical Medicine Research Ethics Committee in the UK (protocol 17-032).

We recruited 165 participants, 66 (40%) of whom were PWH. Of the 66 PWH, 55 (83.3%) were on ART. Ten participants, six of whom were PWH, had indeterminate QFT-Plus results and were excluded from further analysis. Of the remaining 155 individuals, 68 [43.87% [95% confidence interval (CI) 35.92–52.06] had positive QFT-Plus results, indicating that they had LTBI. Subgroup analysis by sex revealed that LTBI was more prevalent among males than females [46 of 88 males (52.27%, 95% CI 41.35–63.04) vs. 22 of 67 females (32.84%, 95% CI 21.85–45.40), *P* = 0.02]. However, the prevalence of LTBI was similar across different age groups (*P* = 0.82, Fig. 1a) and between PWH and HIV-uninfected individuals [48.33% (95% CI 35.23–61.61) vs. 41.05% (95% CI 31.06–51.62), *P* = 0.37].

**Fig. 1:**
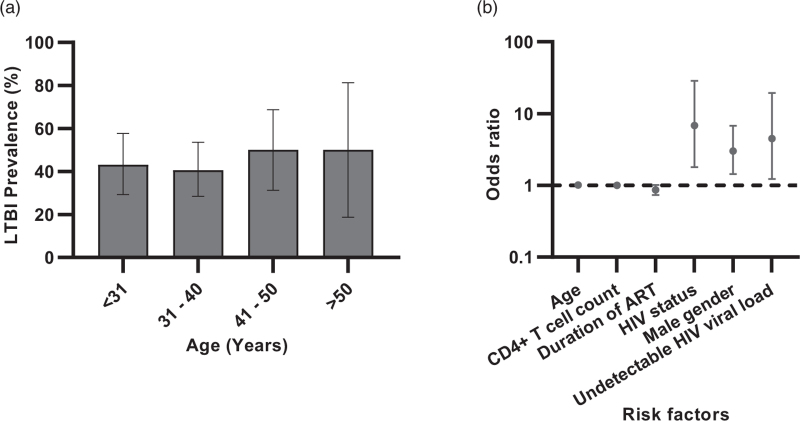
(a) Prevalence of latent tuberculosis infection according to age. (b) Multivariate logistic regression analysis of risk factors of latent tuberculosis infection. Error bars: 95% confidence interval. *N* = 60 HIV-positive; 95 HIV-negative.

Next, we performed multiple logistic regression to determine if LTBI was associated with age, sex, HIV status, plasma HIV viral load or peripheral blood CD4^+^ T-cell count. We found that HIV infection, male sex and undetectable HIV viral load were independent risk factors for LTBI [odds ratio (OR) 6.81, CI 1.79–28.66, *P* = 0.006; OR 3.02, CI 1.41–6.77, *P* = 0.006; OR 4.49, CI 1.23–19.36, *P* = 0.03, respectively, Fig. 1b], while age and CD4^+^ T-cell count were not.

The current study reports a high prevalence of LTBI among adults attending HIV services at an urban hospital in Malawi, a country with high TB and HIV prevalence. Our findings are consistent with previous reports from Nigeria [3] and Zimbabwe [4]. Although HIV infection is a known risk factor for active TB [5], we show that it is also a risk factor for LTBI. The high burden of LTBI among PWH in SSA justifies the WHO recommendation of preventive treatment for LTBI in PWH even in the absence of IGRA or tuberculin skin test results [6].

The high prevalence of LTBI in SSA is likely indicative of high community transmission of TB. Currently, most countries in the region treat PWH with isoniazid preventive therapy (IPT) for 6–36 months [7–9]. Although IPT significantly reduces the risk of LTBI progressing to active TB, its protective effect wanes following discontinuation [10]. Therefore, lifelong IPT would provide PWH with long-lasting protection against active TB. In addition, the high LTBI prevalence among HIV-uninfected adults calls for a review of the public health response to TB in the region. Treatment of LTBI in other at-risk groups should be explored to reduce the reservoir for active TB cases.

We acknowledge that the small sample size and the hospital-based recruitment may limit generalizability of our study findings, therefore large community-based studies are required to determine the community prevalence of LTBI in Malawi. Nonetheless, our participants were ambulatory and reflect the composition of the communities served by the hospital.

In conclusion, the prevalence of LTBI in the study population was high. Males and PWH were at high risk of LTBI. Treating individuals with LTBI in SSA, including lifelong IPT for PWH should complement current active TB case finding strategies to reduce TB incidence and deaths in the region.

## Acknowledgements

H.C.M. conceived and designed the experiments. S.C.M.-N., D.T.M., E.T.C. and A.P.C. performed the experiments and analysed the data. S.C.M.-.N. and H.C.M. wrote the article, with input from D.T.M., E.T.C., A.P.C., D.V.M., C.K., C.M., D.L.T., J.M., L.M., D.G.R., K.C.J. and S.B.S. The authors thank all study participants and staff of the Clinical Investigation Unit, Malawi Liverpool Wellcome Clinical Programme, Kamuzu University of Health Sciences and Queen Elizabeth Central Hospital for their support and co-operation during the study.

The current work was supported by an African Research Leader Award MR/P020526/1 awarded to H.C.M. and S.B.S., jointly funded by the UK Medical Research Council (MRC) and the UK Department for International Development (DFID) under the MRC/DFID Concordant agreement and are part of the EDCTP2 programme supported by the European Union. The work was also supported by the National Institutes of Health (USA) award AI155319 to D.G.R. A core grant 206545/Z/17/Z from Wellcome supports the Malawi Liverpool Wellcome Clinical Research Programme.

### Conflicts of interest

There are no conflicts of interest.
